# Segmental Zoster Paresis of the Unilateral Lower Extremity With Neuritis MRI Findings: A Case Report and Literature Review

**DOI:** 10.7759/cureus.30398

**Published:** 2022-10-17

**Authors:** Hamisi M Mraja, Sule Nur Mraja, Inas Mohamed Fawzy Daadour, Ayhan Mutlu, Selhan Karadereler, Meric Enercan, Azmi Hamzaoglu

**Affiliations:** 1 Orthopedics and Traumatology, Istanbul Florence Nightingale Hospital, Istanbul, TUR; 2 Neurology, Duzce Ataturk State Hospital, Duzce, TUR; 3 Integrative/Complementary Medicine, Istanbul Florence Nightingale Hospital, Istanbul, TUR; 4 Radiology, Istanbul Florence Nightingale Hospital, Istanbul, TUR; 5 Neurological Surgery, Istanbul Florence Nightingale Hospital, Istanbul, TUR; 6 Orthopedics and Traumatology, Demiroglu Bilim University, Istanbul, TUR; 7 Spine Surgery, Istanbul Florence Nightingale Hospital, Istanbul, TUR

**Keywords:** neuritis, mri, radiculopathy, varicella zoster virus, herpes zoster, segmental zoster paresis

## Abstract

Herpes zoster (HZ) is a common clinical condition caused by the reactivation of the latent varicella-zoster virus (VZV). Neurological complications after HZ have been described, including a rare condition of segmental zoster paresis (SZP), which results in unilateral motor impairment in the extremities. Only two cases of HZ patients with radiculopathy and MRI findings of neuritis have been reported. We present a 62-year-old male with a HZ rash in the right calf and low back pain radiating to the right leg accompanied by a right leg great toe weakness for one week. Neurological examination revealed 4/5 dorsiflexion of the right great toe. Also, the patient’s rash was distributed on the L5 dermatome. The lumbar MRI showed a contrast enhancement of the right L5 nerve root with enlargement diagnosed as neuritis. The patient was treated with valacyclovir. The neuromotor deficit and the cutaneous rash started to improve on the third day of treatment. This case emphasizes the necessity of considering SZP in the differential diagnosis of elderly patients presenting with muscle weakness in the lower extremity with or without a rash. MRI evaluations of HZ patients with radiculopathy may include contrast-enhanced sequences.

## Introduction

Herpes zoster (HZ) is associated with numerous complications, including postherpetic neuralgia, encephalitis, aseptic meningitis, and segmental zoster paresis (SZP) [1]. However, SZP, which results in unilateral motor impairment in the extremity, is a rare complication of HZ. This rare condition of SZP may cause misdiagnosis in elderly patients presenting with lower extremity radiculopathy. Only two cases of SZP patients with MRI findings of neuritis have been previously reported. This MRI finding has not been sufficiently elaborated on in the literature. We present a case of a 62-year-old man who was admitted with low back pain radiating to the right leg accompanied by right leg great toe weakness before cutaneous eruptions and developed ipsilateral lower extremity rash after paresis. To our knowledge, this is the third case with SZP, and neuritis recorded with MRI findings.

## Case presentation

**Figure 1:**
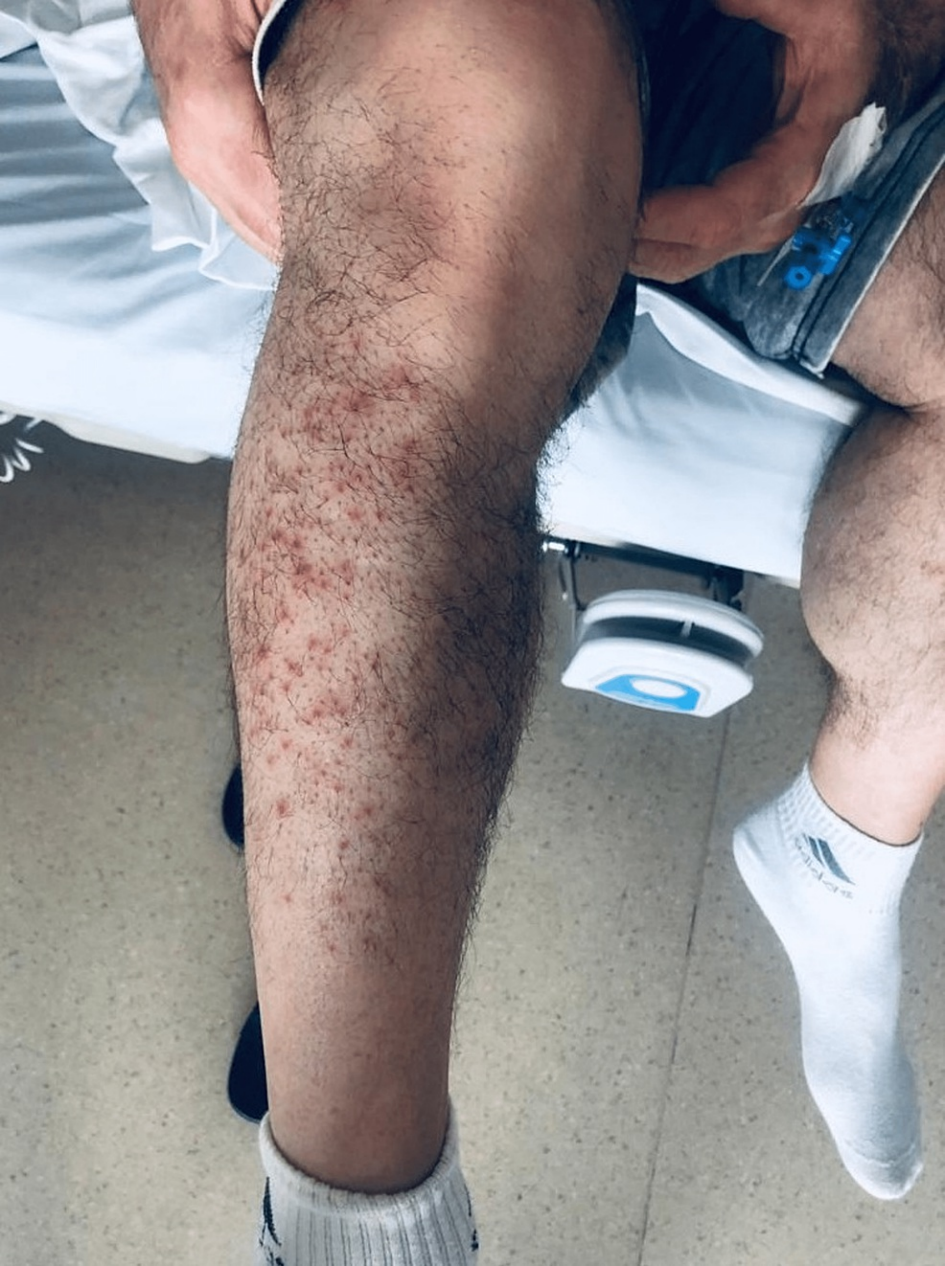
The patient’s cutaneous rash distributed on the L5 dermatome.

A 62-year-old man was admitted to our hospital with acute onset right low back pain radiating to the right leg accompanied by HZ rashes on his right lateral calf and dorsiflexion weakness in the right leg's great toe. The patient's history of HZ rash existed for one week. No history of trauma, allergenic skin disease, or poisoning. Also, the patient was not under any medical prescription. His physical examination elaborated a vesicular erythematous rash in the right lateral calf along the L5 dermatome (Figure 1). Neurological examination revealed only 4/5 dorsiflexion of the right great toe without abnormal deep tendon reflexes (DTR) or any muscle deficit in the other extremities. There were no pathologic reflexes recorded.

**Figure 2:**
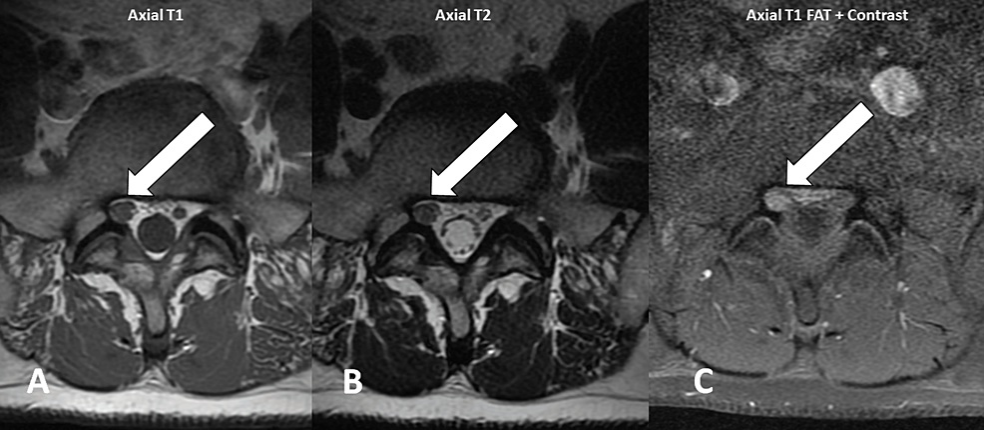
(A-C) MRI images of the lumbar spine demonstrate neuritis of the right L5 nerve root (indicated by the arrow) with contrast enhancement and enlargement.

The patient with radicular pain and neurological deficit was scheduled for a lumbar MRI. We evaluated all the MRI images and identified the right L5 nerve root enlargement on the axial images (Figures 2A, 2B). Contrast-enhanced images were performed additionally. We recorded enhancement of the right L5 nerve root (Figure 2C). This enlargement and enhancement in the contrast images of the right L5 nerve root were described as neuritis.

We performed a literature search with these findings. Only two cases of patients with acute onset HZ rash presenting with radiculopathy of the lower extremity with dermatomal rash and lumbar nerve enhancement on MRI have been described in the literature [1,2]. We performed a consultation with our neuroradiologist team and concluded by suggesting the lesion may be associated with HZ. Afterward, we performed dermatology, and infectious disease consultations and the patient was diagnosed with HZ. With our neuroradiologist team, we concluded the patient’s different diagnosis as being SZP. Valacyclovir treatment of 3X1g dosage was initiated. Clinical improvement was appreciated three days after antiviral treatment for the neuromotor deficit and HZ rash. On discharge, pregabalin was also prescribed and the L5 nerve root lesion was conservatively treated.

## Discussion

Varicella-zoster virus (VZV) causes chickenpox infection and may remain dormant in the body's nerve tissues for years. The virus is usually kept under control by the immune system. However, with time and especially in immunosuppressed or elderly patients, it might cause a different form of viral infection called shingles (also known as HZ or night burn) [3,4]. It is characterized by liquid-filled painful vesicles accompanied by redness most frequently on the trunk, but potentially elsewhere. In shingles, the virus remains hidden in the nerve roots throughout the spinal cord. When the VZV is reactivated, the retrograde spread of the virus results in neuritis [3]. Later, it reaches the skin via the sensory nerve from the dorsal root ganglion, causing a dermatomal rash on one side of the body [3].

After VZV infection, immunocompromise, smoking, and diabetes are mainly associated with neurological complications [5]. Among these neurological complications, SZP is one of the complications that rarely occur in approximately 3%-5% of cases. SZP usually is seen in the elderly group affecting mainly the facial muscles, followed by the upper limb muscles [6-8]. Lower extremity involvement is rare [7,8]. Weakness usually occurs a few days to weeks after the appearance of the herpetic rash and in the proximal muscle groups (C5, C6, C7 or L2, L3, L4 innervated muscles) [6]. Nerve enlargement and T2 hyperintensity consistent with clinical symptoms are rarely seen in MRI examinations of patients with SZP [1,2]. SZP management including antiviral treatment and symptomatic treatments has a good prognosis.

In our patient, there was a low back pain radiating to the right leg accompanied with right leg great toe weakness before cutaneous eruptions and developed ipsilateral lower extremity rash after paresis. It is a rare form of SZP with MRI enhancement of the L5 nerve root resulting in focal weakness in the right lower extremity in its myotome (L5) and accompanied by a corresponding dermatomal (L5) rash. In the literature, only two cases of lower extremity SZP have been reported as having MRI enhancement of the affected nerve root (Table 1) [1,2].

**Table 1:** Review of the literature

Study	Cases	Age Gender	Interval between rash and weakness	Rash distribution	Imaging findings	Factors
Patel et al. 2022 [1]	1	80M	–	Left L3/L4	Enhancement of the left L4 nerve roots	B-cell lymphoma
Bhushan et al. 2020 [7]	1	55W	3 days	Left thigh and upper calf in the L5/S1 region	Enhancement of the left L5 nerve root	–
Our case	1	62M	7 days	Right lateral calf	Enhancement of the right L5 nerve roots	–

The history and physical findings are sufficient to make the HZ diagnosis. In most cases, it is of no use to confirm the diagnosis through laboratory tests. However, laboratory data for VZV can be obtained by direct fluorescent antibody (DFA), polymerase chain reaction (PCR), and Tzanck smear [9]. Low amplitude of compound muscle action potentials (CMAP) and sensory nerve action potentials (SNAP) suggesting motor and sensory axonopathy may be seen in EMG of patients with SZP [1]. Although EMG and PCR were not performed on our patient the diagnosis is set by clinical evaluation.

The pathogenesis behind the development of SZP is still unclear. One theory suggests that a local inflammation around the dorsal root ganglion causes hypervascularity in the perineural structure or disruption of the blood-nerve barrier hence the development of neuromotor deficit. According to another theory, there is a spread to the anterior spinal nerve roots, which eventually results in paresis due to the involvement of anterior horn cells [10]. SZP treatment is the same as HZ management. In the literature, SZP takes weeks to months for the affected limb to fully recover [11]. In this case report, our long follow-up was limited.

## Conclusions

This SZP case involving the lower extremity is among the rare HZ complications. In immunocompromised and elderly patients presenting with lumbar radiculopathy, even in the absence of cutaneous rash, SZP may be included in their differential diagnosis. We recommend radiologic evaluations consisting of MRI with contrast-enhanced sequences in SZP cases. Also, in any radiculopathy patient with MRI findings showing contrast-enhancement and enlargement of lumbar nerve roots, SZP may be the potential differential diagnosis.
